# Experimental Study on Ultra-Precision Polishing of Ti-6Al-4V by Ultraviolet-Induced Nanoparticle Colloid Jet Machining

**DOI:** 10.3390/ma14175014

**Published:** 2021-09-02

**Authors:** Xiaozong Song, Xiaorong Wang, Shun Wang, Shengkai Liu, Shundong Ge

**Affiliations:** School of Mechanical and Electronical Engineering, Lanzhou University of Technology, Lanzhou 730050, China; smilewang1997@gmail.com (X.W.); wangshun1923@163.com (S.W.); shengkailiu520@gmail.com (S.L.); ggsd137@gmail.com (S.G.)

**Keywords:** Ti-6Al-4V, ultra-precision polishing, ultraviolet-induced nanoparticle colloid jet machining, surface quality, surface residual stress

## Abstract

Ti-6Al-4V is widely used in various fields of modern industry, but it is difficult to obtain an ultra-smooth surface of Ti-6Al-4V due to its poor machinability. In this article, ultraviolet-induced (UV-induced) nanoparticle colloid jet machining was utilized to carry out ultra-precision polishing of Ti-6Al-4V to improve the surface quality. The results of infrared differential spectroscopy before and after polishing show that new chemical bonds such as Ti-O-Ti (Al-O-Ti and V-O-Ti) appear on the Ti-6Al-4V workpiece surface, which indicates that the material of Ti-6Al-4V workpiece is removed through the chemical interaction between TiO_2_ nanoparticles and workpiece surface in the process of UV-induced nanoparticle colloid jet machining. The comparison of metallographic structure of Ti-6Al-4V before and after polishing shows that the chemical activity and material removal rate of the primary α phase in Ti-6Al-4V is higher than that of the remnant β phase in UV-induced nanoparticle colloid jet machining, which lead to the well-distributed nano-scale surface peaks and valleys at regular intervals on the polished Ti-6Al-4V workpiece surface. After polishing, the longitudinal residual stress on the surface of Ti-6Al-4V workpiece decreases from 75 MPa to 67 MPa and the transverse stress decreases from 13 MPa to 3 MPa. The surface roughness of Ti-6Al-4V workpiece is reduced from Sa 76.7 nm to Sa 2.87 nm by UV-induced nanoparticle colloid jet machining.

## 1. Introduction

Titanium alloys (most commonly Ti-6Al-4V) have been widely used in aerospace, petrochemical, medical equipment field and other fields in recent years [1,2] due to their strong corrosion resistance, non-toxicity, good biocompatibility and high strength [3]. Titanium alloy is one of the most ideal material for medical equipment and artificial bone joint materials. Current research shows that the surface processing quality of titanium alloys devices has a great influence on their performance [4,5], and the surface roughness of titanium alloys devices will affect the bacterial adhesion on its surface. If the surface roughness of the medical devices is too rough, the bacterial adhesion and infection will be intensified, which can improve the cost and complicate the using process of these medical devices. In addition, too much surface roughness makes the surface wear resistance of titanium alloys implants worse [6,7], which may have a certain impact on human health [8,9]. Therefore, reducing the surface roughness is very important for improving the performance of titanium alloys devices. However, due to the poor machinability and thermal conductivity of titanium alloys, the surface quality of titanium alloy in traditional cutting and grinding is poor, and there is residual stress on the surface of the workpiece [10]. At present, experts and scholars have done lots of research on the surface polishing process of titanium alloys to improve the surface quality. The main polishing methods of titanium alloys are mechanical polishing, electrolytic polishing, abrasive flow polishing, chemical mechanical polishing, ion beam polishing, high energy beam polishing and so on. The surface characteristics of commercially pure titanium parts fabricated by electron beam melting polished by the electrolytic polishing process was studied and the surface quality of commercially pure titanium parts was improved by the electrolytic polishing [11]. The effectiveness of various polishing processes based on chemical, mechanical and electrochemical were investigated to establish the effective methods for smooth Ti surface preparation before anodization. The results showed that chemical mechanical polishing had the best machining effect on the surface of pure titanium, with high surface quality and no obvious surface defects [12]. Laser polishing was used to process the surface of Ti-6Al-4V workpiece, and the surface roughness of the Ti-6Al-4V workpiece was reduced from 5.226 μm to 0.375 μm, the hardness and surface wear resistance of Ti-6Al-4V workpiece were also improved to a certain extent after laser polishing [13].

In this article, the ultra-precision polishing experiments of Ti-6Al-4V were carried out by UV-induced nanoparticle colloid jet machining [14,15] to obtain an ultra-smooth workpiece surface. Under the action of ultraviolet light, the chemical interaction between the nanoparticles and the surface atoms of the workpiece [16] was utilized to remove the surface materials of Ti-6Al-4V at the atomic scale. The material removal mechanism of Ti-6Al-4V in UV-induced nanoparticle colloid jet machining was investigated by Fourier-transform infrared spectroscopy (FT-IR). The surface roughness and micro surface morphology of Ti-6Al-4V were analyzed, and the surface residual stress was investigated to evaluate the processing ability of UV-induced nanoparticle colloid jet machining on Ti-6Al-4V. The corresponding reason for the well-distributed surface peaks and valleys at regular intervals on the Ti-6Al-4V workpiece surface after being polished were also discussed through metallographic structure and atomic force microscope (AFM) analysis.

## 2. Materials and Methods

**Figure 1:**
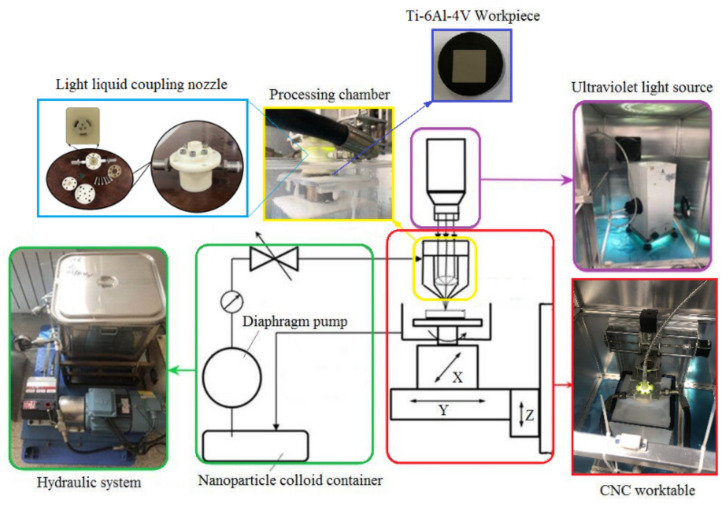
Experimental equipment of UV-induced nanoparticle colloid jet machining.

According to the principle of UV-induced nanoparticle colloid jet machining [17,18], the nanoparticle colloid needs to form a continuous and stable jet beam with certain kinetic energy through the light liquid coupling nozzle under the action of pressure, and the colloid jet beam impacts the workpiece surface to achieve the desired machining purpose. Therefore, the UV-induced nanoparticle colloid jet machining equipment is mainly composed of pressure system, light source system and processing system. Figure 1 shows the schematic diagram of the experimental equipment of UV-induced nanoparticle colloid jet machining. All the experiments in this paper were carried out on the experimental equipment of UV-induced nanoparticle colloid jet machining, which was independently developed by the research group.

**Figure 2:**
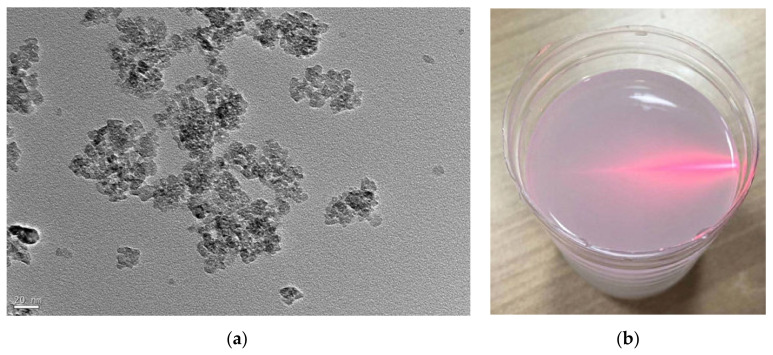
(**a**) TEM morphology of TiO_2_ nanoparticles; (**b**) colloidal slurry samples for polishing experiments.

In the polishing experiments, anatase TiO_2_ was utilized as the polishing abrasive. Figure 2a shows the TEM morphology image of the anatase TiO_2_ nanoparticles, which was carried out using a JEM-1200EX transmission electron microscope (TEM, Jeol Company, Toyoshima, Tokyo, Japan). According to the TEM morphology results, the anatase TiO_2_ nanoparticles have flat and irregular sheet shapes, and the diameters of TiO_2_ nanoparticles are about 20–30 nm. The anatase TiO_2_ nanoparticles were uniformly dispersed in deionized water to prepare the colloidal polishing solution as shown in Figure 2b. The pH value of the colloidal polishing solution was adjusted by organic malic acid. The density of the colloidal polishing solution with 10% (volume concentration) TiO_2_ nanoparticles is about 1200 kg/m^3^, and the viscosity is about 1.3 mPa·s at room temperature. Table 1 shows the relevant polishing experimental parameters in this study.

**Figure 3:**
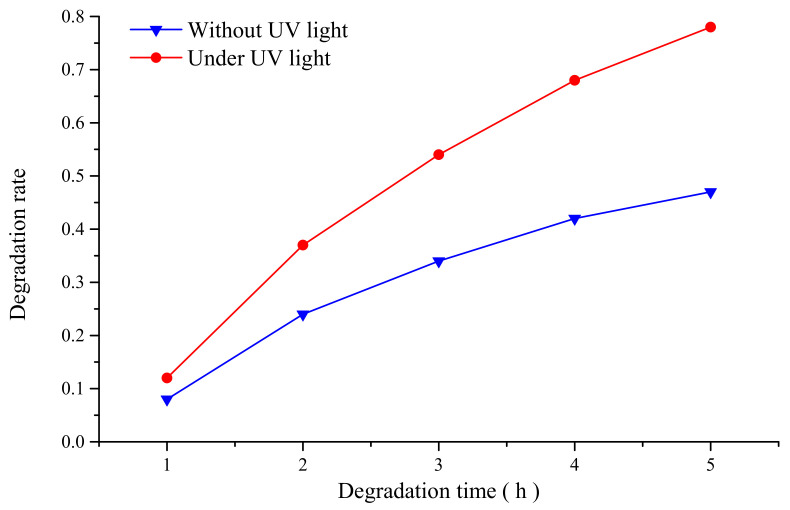
Comparison of methyl orange degradation by TiO_2_ nanoparticle colloid.

In order to determine the photocatalytic activity of the colloidal polishing solution, the degradation experiments of methyl orange solution were carried out. About 0.01 g methyl orange powder (AR, Comeo, Tianjin, China) was weighed by electronic balance, and 0.001 g/L methyl orange solution was obtained by dissolving the methyl orange powder in 1000 mL distilled water with a glass rod, and then the methyl orange solution was diluted 100 times before using. About 400 mL diluted methyl orange solution was divided into two equal parts and placed in two open containers, and then 20 mL prepared TiO_2_ nanoparticle colloid was dropped into two beakers with a rubber tipped dropper. After fully stirring with a glass rod, one beaker was placed without UV light and the other beaker was placed under UV light with the intensity of 145 mW/cm^2^. The degradation time was 5 h. About 30 mL solution was removed from the above two solutions per hour and numbered. After degradation for 5 h, 10 samples of solution were obtained and detected by UV-Vis spectrophotometer (U-2001, Hitachi, Tokyo, Japan). The absorption curve of ten samples and the absorbance at the maximum wavelength are substituted into the following degradation rate formula:

(1) $$x=\frac{A_{0}-A_{t}}{A_{0}}\times 100\%,$$

where *x* is the degradation rate, *A*_0_ stands for the absorbance of the sample without photocatalytic degradation, and *A_t_* is the absorbance after degradation. According to the degradation rate calculated by the above formula, the degradation rate curve was drawn as shown in Figure 3.

According to the above comparison results, the degradation rate of methyl orange solution by TiO_2_ nanoparticle colloid under UV light is significantly higher than that of methyl orange solution without UV light, which indicates that the prepared TiO_2_ nanoparticle colloid polishing solution has good photocatalytic activity under UV light. However, with the degradation time getting longer, the degradation speed of methyl orange gradually decreases with time.

The Ti-6Al-4V workpieces before and after UV-induced nanoparticle colloid jet machining were removed and soaked in deionized water for about 10 s, and then these Ti-6Al-4V workpieces were dried naturally at room temperature. The Fourier-transform infrared spectroscopy (FT-IR, Nexus 670 FT-IR spectrometer, Nicoli, Madison, WI, USA) was utilized to characterize the above prepared Ti-6Al-4V workpiece.

In order to accurately measure the surface roughness and surface micro morphology, the Ti-6Al-4V workpieces before and after UV-induced nanoparticle colloid jet machining were rinsed with a pure water jet to completely remove the remaining nanoparticles and other surface contamination. A portable surface roughness measuring instrument (SJ-410, Mitutoyo, Kanagawa, Japan) was used to measure the linear rugosity of Ti-6Al-4V workpiece before and after being polished with the measurement length of 0.4mm. In order to obtain more comprehensive surface roughness and morphology information of the Ti-6Al-4V workpiece before and after UV-induced nanoparticle colloid jet machining, a noncontact three-dimensional surface profilometer (MicroXAM-800, KLA-Tencor, Milpitas, CA, USA) was also employed to characterize the surface morphology and roughness of the Ti-6Al-4V workpiece in different measuring areas.

In this paper, the X-ray diffraction method was used to measure the surface residual stress. The testing instrument was X-ray residual stress instrument (μ-X360s X-ray residual stress analyzer, Pulstec, Hamamatsu, Shizuoka, Japan). A beam of X-ray irradiated on the Ti-6Al-4V sample through the middle hole of the flat panel detector, and the detector obtained the Debye ring by recording the ring information of the diffraction peak. Additionally, then the distribution of Debye ring was calculated by computer, and the surface residual stress of the sample was obtained [19,20]. During the measurement, the X-ray incidence angle was 25.0 degree and the sample distance was 33.7 mm.

Metallographic analysis of Ti-6Al-4V workpiece before and after polishing were carried out by using a metallographic microscope (GX51, Olympus, Tokyo, Japan). Additionally, the surface micro morphology of Ti-6Al-4V workpieces were measured by AFM (Bruker Dimension Icon, Bruker Corporation, Karlsruhe, Germany) with a measurement area of 10μm × 10μm. All the measuring area were randomly selected on the Ti-6Al-4V workpiece in this study.

## 3. Results

### 3.1. Interaction between TiO_2_ Nanoparticles and Ti-6Al-4V Workpiece

According to the material removal mechanism of UV-induced nanoparticle colloid jet machining, a large number of hydroxyl (OH) groups produced in the TiO_2_ colloid under the action of photocatalysis of ultraviolet light [21] can interact with the atoms on the surface of the TiO_2_ nanoparticles and Ti-6Al-4V workpiece to generate surface hydroxyl groups. In UV-induced nanoparticle colloid jet machining, when the TiO_2_ nanoparticles impact on the Ti-6Al-4V workpiece, an interfacial chemical reaction may take place between the TiO_2_ nanoparticles and the Ti-6Al-4V surface, in which new chemical bonds (such as Ti-O-Ti, Al-O-Ti and V-O-Ti bonds) and products are generated.

(Al, V)Ti—OH + OH—Ti → (Al, V)Ti—O—Ti + H_2_O (Ch), (2)

where H_2_O (CH) is a chemically adsorbed water molecule.

**Figure 4:**
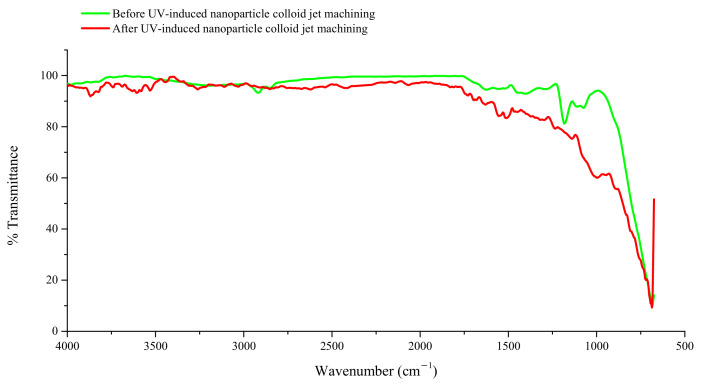
FT-IR of Ti i-6Al i-4V before and after UV-induced nanoparticle colloid jet machining.

**Figure 5:**
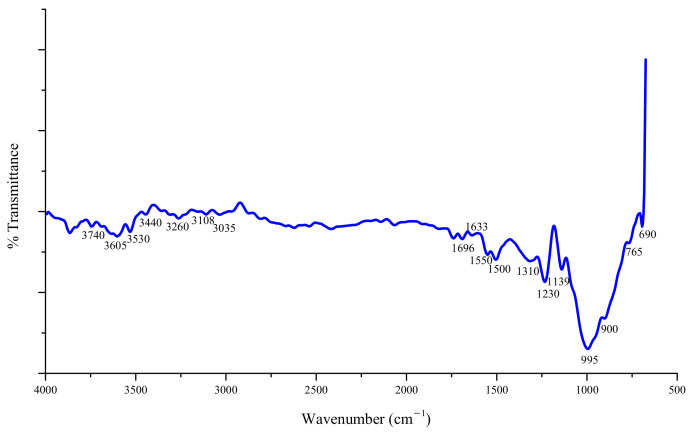
The FT-IR differential spectra of the Ti-6Al-4V workpieces.

Figure 4 shows the FT-IR spectra of the Ti-6Al-4V workpiece surface. The FT-IR differential spectra was obtained by deducting the original bands of the Ti-6Al-4V workpiece surface to show the differences in the infrared spectra of the Ti-6Al-4V surface before and after UV-induced nanoparticle colloid jet machining. Figure 5 displays the FT-IR differential spectra to show the new bands generated in the process of polishing. As the Ti-6Al-4V workpiece was only dried naturally after being polished in UV-induced nanoparticle colloid jet machining, the infrared absorption peaks of free water inevitably appear in the infrared spectrum. The band at 3740 cm^−1^ is attributed to the stretching vibration of the free OH group, and the band at 1550 cm^−1^ is attributed to the bending vibration of the free OH group. The bands at 3605, 3530 and 3440 cm^−1^ are attributed to the stretching vibration of the OH groups adsorbed on the Ti-6Al-4V surface in various forms, and the bands 1696, 1633, 1310 and 1230 cm^−1^ belong to the bending vibration of the corresponding OH groups [21,22]. These results indicate that there are a large number of OH groups adsorbed on the Ti-6Al-4V surface in various forms during the process of UV-induced nanoparticle colloid jet machining. The band at 690 cm^−1^ belongs to the Ti-O band [23,24,25], indicating that there are TiO_2_ nanoparticles adsorbed on Ti-6Al-4V workpiece. The bands at 765 cm^−1^ belong to the Al-O band. In the frequency region of the M-O-M group (900–1500 cm^−1^), the new bands at 900 cm^−1^, 995 cm^−1^ and 1139 cm^−1^ are correspondingly attributed to the V-O-Ti, Ti-O-Ti and Al-O-Ti stretching vibration bands [26], which indicate that there are new generated chemical bonds and chemical reactions between TiO_2_ nanoparticles and Ti-6Al-4V workpiece surface in the process of UV-induced nanoparticle colloid jet machining. Additionally, under the shear viscosity of colloid jet, the TiO_2_ nanoparticles adsorbed on the Ti-6Al-4V surface will be taken away from the workpiece together with the atoms of the Ti-6Al-4V surface, and then, realizing the ultra-precision machining of the Ti-6Al-4V workpiece.

### 3.2. Surface Quality of Ti-6Al-4V Polished by UV-Induced Nanoparticle Colloid Jet Machining

#### 3.2.1. Surface Roughness and Micro Surface Morphology of Ti-6Al-4V

**Figure 6:**
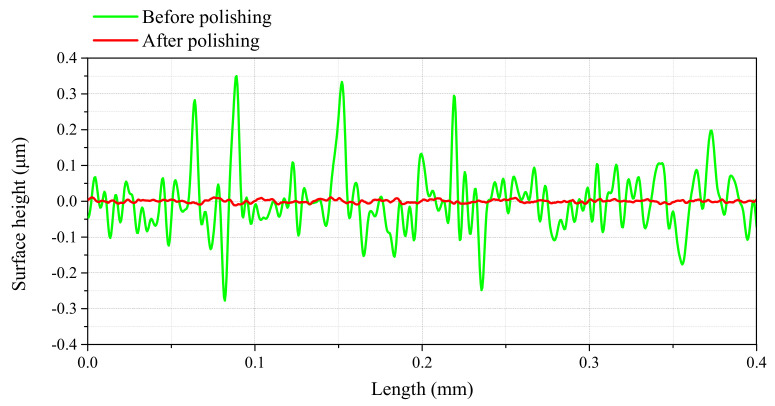
The contour curve of Ti-6Al-4V workpiece surface before and after polishing.

The contour curve of Ti-6Al-4V workpiece surface before and after polishing are shown in Figure 6, and the roughness value of linear rugosity are shown in Table 2. The results show that the surface roughness and the maximum height difference of Ti-6Al-4V workpiece have been greatly reduced after UV-induced nanoparticle colloid jet machining. The surface roughness Ra was reduced from 66 nm to 3 nm, Rq was reduced from 93 nm to 4 nm, and the maximum height difference Rz was reduced from 662 nm to 18 nm.

**Figure 7:**
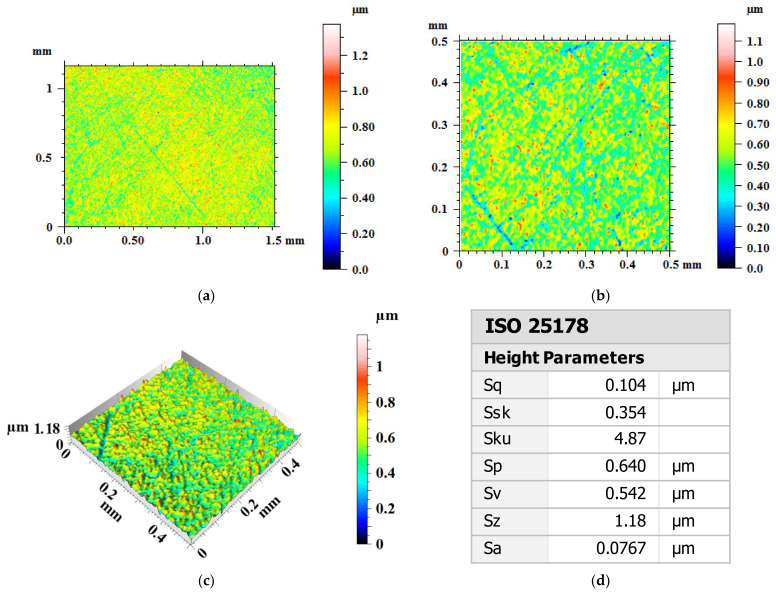
Surface morphology and roughness of Ti-6Al-4V workpiece before polishing. (**a**) Two-dimensional surface topography with measuring area of 1.5 mm × 1.15 mm; (**b**) two-dimensional surface topography with measuring area of 0.5 mm × 0.5 mm; (**c**) three-dimensional surface topography with measuring area of 0.5 mm × 0.5 mm (**d**) surface roughness results.

Figure 7 shows the surface morphology and roughness of the Ti-6Al-4V workpiece before polishing. Figure 7a is the two-dimensional surface topography with measuring area of 1.5 mm × 1.15 mm. The crisscross line like scratch marks on the Ti-6Al-4V workpiece surface can be seen clearly in the figure. Figure 7b,c show the two-dimensional and three-dimensional surface topography with measuring area of 0.5 mm × 0.5 mm. A large number of randomly distributed surface bumps and grooves can be found on the original Ti-6Al-4V surface with peak-to-valley height (PV) of 1.18 μm. The surface roughness values of the original Ti-6Al-4V within the measuring area of 0.5 mm × 0.5 mm are of Sa 76.7 nm (0.0767 μm) and Sq 104 nm (0.104 μm).

**Figure 8:**
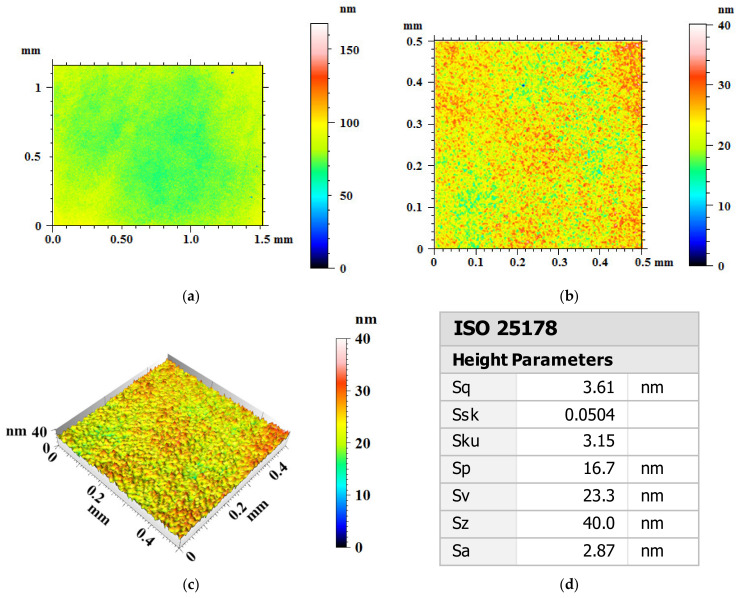
Surface morphology and roughness of Ti-6Al-4V workpiece after polishing. (**a**) Two-dimensional surface topography with measuring area of 1.5 mm × 1.15 mm; (**b**) two-dimensional surface topography with measuring area of 0.5 mm × 0.5 mm; (**c**) three-dimensional surface topography with measuring area of 0.5 mm × 0.5 mm; (**d**) surface roughness results.

After 180 min polishing by UV-induced nanoparticle colloid jet machining, the surface morphology and roughness of the Ti-6Al-4V workpiece were shown as Figure 8. As a result of polishing, the crisscross line like scratch marks on the original Ti-6Al-4V workpiece surface have been removed from the Ti-6Al-4V workpiece surface as shown in Figure 8a. After being polished, there are well-distributed nano-scale surface peaks and valleys at regular intervals on the polished Ti-6Al-4V workpiece surface with peak-to-valley height (PV) of 40nm as shown in Figure 8b,c. Table 3 shows the surface roughness values of random measuring points on each workpiece surface. The surface roughness values of the Ti-6Al-4V within the measuring area of 0.5 mm × 0.5 mm are of Sa 2.87 nm and Sq 3.61 nm.

**Figure 9:**
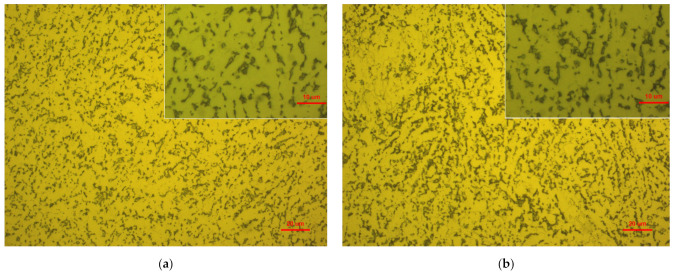
Metallographic structure of Ti-6Al-4V before and after polishing. (**a**) Before polishing. (**b**) After polishing.

In order to study the reason for the formation of these well-distributed nano-scale surface peaks and valleys at regular intervals, metallographic analysis of Ti-6Al-4V workpiece before and after polishing were carried out by using a metallographic microscope as shown in Figure 9. Ti-6Al-4V belongs to α + β phase titanium alloy, which is formed by adding a small amount of β phase stable elements in α phase titanium alloy [27]. In the metallographic structure diagram shown in Figure 9, the lighter part is the primary structure of close packed hexagonal structure α phase, and the darker part is the body centered hexagonal structure β phase. It can be observed that the metallographic characteristics of the two Ti-6Al-4V workpieces before and after polishing are the same, the remnant β phase distributed around the thick and light colored primary α phase. The arrangement of the two phases is equiaxed. The microstructure of Ti-6Al-4V cannot be changed by chemical adsorption and shear viscosity removal process in UV-induced colloidal jet machining. When comparing Figure 9a with Figure 9b, it can be found that there is no change in the arrangement of the primary α and remnant β phases on the two Ti-6Al-4V workpieces surface before and after UV-induced colloidal jet machining, but the area proportion of remnant β phase on the Ti-6Al-4V workpieces surface after polishing is larger than that of before polishing. The reason for this result is mainly due to that in Ti-6Al-4V the chemical activity of primary α phase is higher than that of remnant β phase, and the microhardness of primary α phase is lower than that of the remnant β phase [28]. In the process of UV-induced nanoparticle colloid jet machining, the TiO_2_ nanoparticles in the polishing solution are more likely to interact with the primary α phase, after the interfacial chemical reaction, the material of the primary α phase with lower hardness is easier to remove from the workpiece. In other words, the material removal rate of the primary α phase is higher than that of the remnant β phase in UV-induced nanoparticle colloid jet machining. As a result, on the polished Ti-6Al-4V workpiece surface the microscopic height of the remnant β phase is higher than that of the primary α phase. This makes the area proportion of the remnant β phase on the polished Ti-6Al-4V workpiece surface is more than that before polishing. This also can lead to the well-distributed nano-scale surface peaks and valleys at regular intervals on the polished Ti-6Al-4V workpiece surface as shown in Figure 8.

**Figure 10:**
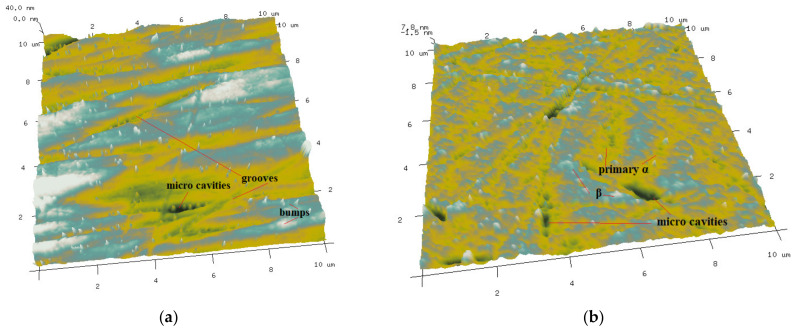
AFM surface micro morphology of Ti-6Al-4V before and after polishing. (**a**) Before polishing. (**b**) After polishing.

In order to clarify the difference of surface micro morphology of Ti-6Al-4V before and after polishing in a smaller scale, the surface morphology of the Ti-6Al-4V workpieces were measured by AFM with a measurement area of 10 μm × 10 μm. Figure 10 shows the AFM surface micro morphology of the Ti-6Al-4V workpieces before and after polishing. The surface of original Ti-6Al-4V workpiece before polishing is covered with a large number of surface bumps and grooves produced by mechanical scraping. In addition, there are some randomly distributed micro cavities, which are inherent defects formed during smelting in Ti-6Al-4V. After polishing, the surface bumps and grooves produced by mechanical scraping have been obviously removed from the Ti-6Al-4V workpiece; however, there are micro peaks, valleys and micro cavities on the polished Ti-6Al-4V workpiece surface. From the distribution morphology, the surface morphology of micro peaks and valleys is very similar to the distribution of the primary α and remnant β phases. Therefore, it can be inferred that the surface micro peaks and valleys are caused by the higher removal rate of the primary α phase than the remnant β phase in the UV-induced nanoparticle colloid jet machining. On the surface of Ti-6Al-4V before polishing the mechanical scraping makes part of the micro cavities covered. The covered micro cavities gradually appear after polishing, so that the micro cavities after polishing are more than before polishing on the Ti-6Al-4V workpieces surface.

#### 3.2.2. Surface Residual Stress Investigation of Ti-6Al-4V Workpiece

**Figure 11:**
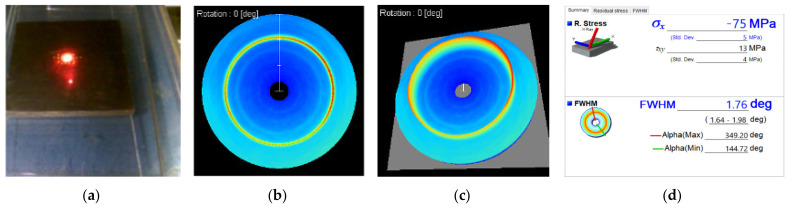
Surface residual stress test diagram and test results of Ti-6Al-4V before polishing. (**a**) Test diagram; (**b**) Debye ring(2D); (**c**) Debye ring(3D); (**d**) surface residual stress.

**Figure 12:**
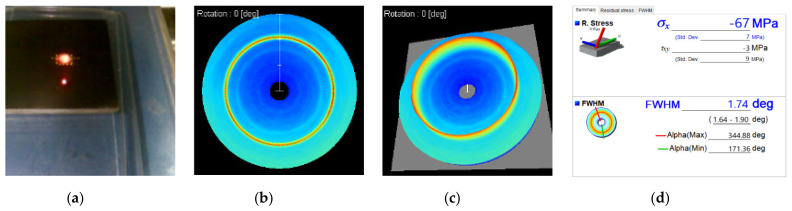
Surface residual stress test diagram and test results of Ti-6Al-4V after polishing. (**a**) Test diagram; (**b**) Debye ring(2D); (**c**) Debye ring(3D); (**d**) surface residual stress.

Residual stress widely exists in products manufactured by machine. Residual stress is generally harmful, which will affect the performance and service life of products. In order to improve the performance of titanium alloys devices, residual stress should be avoided as far as possible during the surface processing of titanium alloys devices. Figure 11 and Figure 12 show the testing process and measurement results of surface residual stress of two Ti-6Al-4V workpieces before and after polishing.

For the general workpiece, the rolling contact load in the manufacturing process determines the longitudinal and transverse stress distribution of the surface area. In the process of rolling and other deformation of Ti-6Al-4V, with the change of grain shape, the overall shape changes and a large number of dislocations are produced. According to the above measurement results, the longitudinal residual stress σ_x_ of Ti-6Al-4V samples is −75 MPa before polishing, and the longitudinal residual stress decreases to −67 MPa after being polished by UV-induced nanoparticle colloid jet machining. The transverse stress of the sample decreases from 13 MPa to −3 MPa, the change of transverse stress direction is caused by the placement direction of titanium alloy sample on the workbench during the measurement.

## 4. Discussion

In the removal process of Ti-6Al-4V by UV-induced nanoparticle colloid jet machining, the results of infrared differential spectroscopy before and after processing show that there are new generated chemical bonds of Ti-O-Ti (Al-O-Ti and V-O-Ti) that appear on the Ti-6Al-4V surface after UV-induced nanoparticle colloid jet machining, which confirm that the interfacial chemical reaction between the TiO_2_ nanoparticles and the Ti-6Al-4V surface was utilized to remove the surface material of Ti-6Al-4V workpiece at atomic level.

After 180 min polishing of UV-induced nanoparticle colloid jet machining, the surface roughness values of the Ti-6Al-4V within the measuring area of 0.5 mm × 0.5 mm have been reduced from Sa 76.7 nm (Sq 104 nm) to Sa 2.87 nm (Sq 3.61 nm). According to the results, there are well-distributed surface peaks and valleys at regular intervals on the polished Ti-6Al-4V workpiece surface. The reason for this phenomenon is that the chemical activity and material removal rate of different phases in Ti-6Al-4V are different, which leads to the high surface roughness of Ti-6Al-4V workpiece after polishing. In reference [29], when Ti-6Al-4V was polished by chemistry enhanced shear thickening polishing with different pH values, well-distributed surface peaks and valleys at regular intervals with the same size (about 5 μm) and height (about 10 nm) could be observed on the workpiece surface. In reference [30], the SEM images of Ti-6Al-4V specimen D, which is polished by chemical polishing, showed the α phase base and β phase on the workpiece surface. The AFM micrographs of specimen D showed more undulate than other specimen that polished by non-chemical polishing methods. The above results of references [29,30] also prove that when the Ti-6Al-4V is polished by chemical polishing, the material removal rate of α phase and β phase of Ti-6Al-4V are different in the process of chemical polishing, and the micro peaks and valleys are left on the surface of the workpiece.

The Ti-6Al-4V workpiece used in this experiment is cut from rolled plate. The longitudinal residual stress is widely distributed in the whole workpiece, and the transverse stress is more distributed on the surface of the workpiece. In the UV-induced nanoparticle colloid jet machining, only a very thin layer of Ti-6Al-4V material was removed from the workpiece surface; therefore, the variation amplitude of the transverse stress is larger than that of longitudinal residual stress.

## 5. Conclusions

In this study, ultra-precision polishing experiments have been done in UV-induced nanoparticle colloid jet machining to improve the surface quality of Ti-6Al-4V. Anatase TiO_2_ nanoparticles with diameters of 20–30 nm were uniformly dispersed in deionized water to prepare the colloidal polishing solution. The degradation experiment of methyl orange indicates that the prepared TiO_2_ nanoparticle colloid polishing solution has good photocatalytic activity under UV light. The FT-IR differential spectra results show that new chemical bonds such as Ti-O-Ti (Al-O-Ti and V-O-Ti) appear on the Ti-6Al-4V workpiece surface, which confirm that chemical reaction exists between TiO_2_ nanoparticles and Ti-6Al-4V surface atoms during the process of UV-induced nanoparticle colloid jet machining. The polishing results show that the crisscross line like scratch marks on the original Ti-6Al-4V workpiece have been removed, and the surface roughness values of the Ti-6Al-4V have been reduced from Sa 76.7 nm (Sq 104 nm) to Sa 2.87nm (Sq 3.61 nm). However, the material removal rate of primary α phase in Ti-6Al-4V is higher than that of the remnant β phase in UV-induced nanoparticle colloid jet machining, which leads to the well-distributed nano-scale surface peaks and valleys at regular intervals on the polished Ti-6Al-4V workpiece surface. The residual stress measurement results show that UV-induced nanoparticle colloid jet machining is a chemical polish method, which can significantly reduce the residual stress in the shallow layer of the workpiece surface.

## Figures and Tables

**Table 1:** Relevant parameters in the polishing experiments.

Experimental Conditions	Value
Workpiece material	Ti-6Al-4V
Workpiece size	25 mm × 25 mm × 1.5 mm
Nanoparticles material	TiO_2_
Diameter of nanoparticles	20–30 nm
Concentration of colloid	10% (volume percentage)
pH of colloid	5
UV light intensity	70 mW/cm^2^
Intensity of pressure	3 Mpa
Polishing time	180 min
Injection distance	3 mm

**Table 2:** Linear rugosity of the Ti-6Al-4V workpieces before and after polishing.

Surface Roughness	Ra (nm)	Rq (nm)	Rz (nm)
Before polishing	66	93	662
After polishing	3	4	18

**Table 3:** Areal rugosity of the Ti-6Al-4V workpieces before and after polishing.

Surface Roughness	Sa (nm)	Sq (nm)	Sz (nm)
Before polishing	76.7	104	1180
After polishing	2.87	3.61	40

## Data Availability

Not applicable.
